# High‐Performance Anion Exchange Membrane Water Electrolyzers Enabled by Highly Gas Permeable and Dimensionally Stable Anion Exchange Ionomers

**DOI:** 10.1002/advs.202402969

**Published:** 2024-06-03

**Authors:** Fanghua Liu, Kenji Miyatake, Masako Tanabe, Ahmed Mohamed Ahmed Mahmoud, Vikrant Yadav, Lin Guo, Chun Yik Wong, Fang Xian, Toshio Iwataki, Makoto Uchida, Katsuyoshi Kakinuma

**Affiliations:** ^1^ Clean Energy Research Center University of Yamanashi Kofu Yamanashi 4008510 Japan; ^2^ Research Organization for Nano and Life Innovation Waseda University Tokyo 1698555 Japan; ^3^ Hydrogen and Fuel Cell Nanomaterials Center University of Yamanashi Kofu Yamanashi 4008510 Japan; ^4^ Department of Applied Chemistry Waseda University Tokyo 1698555 Japan

**Keywords:** anion exchange ionomers, anion exchange membrane water electrolyzers, dimensional stability, high gas permeability, in situ durability

## Abstract

Designing suitable anion exchange ionomers is critical to improving the performance and in situ durability of anion exchange membrane water electrolyzers (AEMWEs) as one of the promising devices for producing green hydrogen. Herein, highly gas‐permeable and dimensionally stable anion exchange ionomers (QC6xBA and QC6xPA) are developed, in which bulky cyclohexyl (C6) groups are introduced into the polymer backbones. QC6_50_BA‐2.1 containing 50 mol% C6 composition shows 16.6 times higher H_2_ permeability and 22.3 times higher O_2_ permeability than that of QC6_0_BA‐2.1 without C6 groups. Through‐plane swelling of QC6_50_BA‐2.1 decreases to 12.5% from 31.1% (QC6_0_BA‐2.1) while OH^−^ conductivity slightly decreases (64.9 and 56.2 mS cm^−1^ for QC6_0_BA‐2.1 and QC6_50_BA‐2.1, respectively, at 30 °C). The water electrolysis cell using the highly gas permeable QC6_50_BA‐2.1 ionomer and Ni_0.8_Co_0.2_O in the anode catalyst layer achieves two times higher performance (2.0 A cm^−2^ at 1.69 V, IR‐included) than those of the previous cell using in‐house ionomer (QPAF‐4‐2.0) (1.0 A cm^−2^ at 1.69 V, IR‐included). During 1000 h operation at 1.0 A cm^−2^, the QC6_50_BA‐2.1 cell exhibits nearly constant cell voltage with a decay rate of 1.1 µV h^−1^ after the initial increase of the cell voltage, proving the effectiveness of the highly gas permeable and dimensionally stable ionomer in AEMWEs.

## Graphical abstract

Terpolymers containing cyclohexyl groups, partially fluorinated aromatic groups, and pendent ammonium tethered fluorenyl groups are designed as anion conductive ionomers. The thin membranes of the terpolymers exhibit high gas permeability as well as hydroxide ion conductivity, and mechanical and chemical robustness. The optimized terpolymer as electrode binders contributes to high‐performance and in situ durability in anion exchange membrane water electrolyzers.

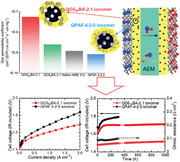

## 1 Introduction

Anion exchange membrane water electrolyzers (AEMWEs) are an attractive electrochemical device for efficient hydrogen production contributing to carbon neutrality. AEMWEs feature lower costs than proton exchange membrane water electrolyzers (PEMWEs) due to the possible use of non‐precious metal catalysts and higher hydrogen production efficiency than alkaline water electrolyzers (AWEs) due to thin electrolyte membranes.^[^ ^1^, ^2^, ^3^, ^4^, ^5^, ^6^, ^7^ ^]^ Compared with already commercialized PEMWEs and AWEs, AEMWEs lags behind and have only developed rapidly in recent years.^[^ ^8^, ^9^, ^10^, ^11^, ^12^, ^13^ ^]^ Enapter is one of the successfully commercialized AEMWEs that produces ca. 1 kg of hydrogen per day using 1 wt.% KOH aqueous solution.^[^ ^14^ ^]^ The operational lifetime of the Enapter's stack system containing 23 cells is claimed to be longer than 35 000 h.^[^ ^15^ ^]^

Some AEMs are commercially available for AEMWEs, such as poly(arylene ether)s with quaternary ammonium groups (FAA‐3‐50), poly(vinylbenzyl methylimidazolium‐*co*‐stryrene)s (Sustanion 37–50), and poly(aryl piperidinium)s (PiperION).^[^ ^16^, ^17^, ^18^ ^]^ In fact, AEMWE using FAA‐3‐50 and precious metal catalysts fed with 1 M KOH aqueous solution exhibited 1.5 A cm^−2^ of the current density at 1.9 V of the cell voltage and 70 °C.^[^ ^16^ ^]^ However, aryl ether groups in the FAA‐3‐50 backbone were susceptible to nucleophilic attack by hydroxide ions, resulting in serious degradation of the membrane and short AEMWEs lifetime. Therefore, most of the recent AEMs do not carry aryl ether groups in the main chain for durability concerns.^[^ ^19^, ^20^, ^21^, ^22^, ^23^, ^24^ ^]^

Anion exchange ionomers (AEIs) being used as electrode binders are another crucial component that also impact much on the performance and long‐term in situ durability of AEMWEs. While a number of research have been devoted to the development of AEMs, AEIs have not been well‐explored.^[^ ^25^, ^26^ ^]^ Most of the AEMWEs utilized the same anion conductive polymers for AEMs and AEIs although different roles are required for each component.^[^ ^27^, ^28^ ^]^ More specifically, in addition to the hydroxide ion conductivity and alkaline stability, AEIs play several other roles such as binding catalyst nanoparticles in the catalyst layers which need to be attached strongly to the AEMs and porous transport layers (PTLs) for efficient electrochemical reactions, where a large amount of product gases (H_2_ and O_2_) are evolved at high current density.^[^ ^29^, ^30^ ^]^ Hyun and co‐workers claimed that the detachment of IrO_2_ nanoparticles from the catalyst layer and the delamination of the catalyst layer from the PTL induced the initial performance degradation of their AEMWEs using polycarbazole‐based (QPC‐TMA) AEIs.^[^ ^31^ ^]^ Replacing QPC‐TMA with proton conductive ionomer (Nafion) improved the in situ durability of AEMWEs with a small voltage decay (35 mV h^−1^ for QPC‐TMA based AEMWE) during the initial 15 h, resulting from the larger binding energy of the binder onto IrO_2_ surface (−100.0 and −43.5 kcal mol^−1^ for Nafion and QPC‐TMA, respectively). Many other studies indicated that the highly hydrophobic (or low ion exchange capacity (IEC)) and dimensionally stable AEIs were favorable for enhancing the ionomer‐catalyst adhesion, in particular, for the anode catalyst layers.^[^ ^32^, ^33^ ^]^ Chen and co‐workers found that decreased swelling of AEIs was effective in prolonging the in situ durability of the resulting AEMWEs.^[^ ^34^ ^]^

Unlike AEMs that should be gas impermeable, AEIs need to be highly gas permeable to discharge rapidly product gases (H_2_ and O_2_) from the catalyst surfaces to mitigate mass transport overpotential.^[^ ^35^, ^36^ ^]^ For improving the gas permeability, increasing microporosity or free volume in AEIs have been investigated. Hu and co‐workers successfully enhanced the gas permeability of AEIs by introducing rigid‐twisted spirobisindane (SPB) components in the polymer backbone.^[^ ^37^ ^]^ As increasing the content of SPB from 10% to 40%, the H_2_ permeability increased while the highest permeability was still lower than that of the commercial proton conductive Nafion. Compared with the platinum group metals (PGM)‐AEMWE with low gas permeability AEI as anode and cathode catalyst layers which achieved 11.17 A cm^−2^ at 2.0 V and 80 °C, AEMWE with higher gas permeability AEI demonstrated better performance (≈1.19 times higher in performance with 13.28 A cm^−2^ at 2.0 V) and in situ durability at 1.0 A cm^−2^ for 1000 h. Fu and co‐workers developed quaternized poly(phenylene‐alkane)s with microporous structures and applied them as anode binder in AEMWE using precious metal catalysts and 1 m NaOH/KOH aqueous solution at 80 °C to achieve 1.28 A cm^−2^ at 1.8 V, which was 1.22 times greater than that of AEMWE using anode AEI with similar IEC but no microporous structure (1.04 A cm^−2^ at 1.8 V).^[^ ^38^ ^]^ After regulating the microporous structure and IEC, the optimal anode ionomer‐based AEMWE reached 1.69 A cm^−2^ at 1.8 V under the same operation conditions.

We have recently reported that the partially fluorinated polyphenylene‐based copolymer AEMs exhibited high hydroxide ion conductivity and chemical stability, making it suitable for AEMWEs.^[^ ^39^, ^40^ ^]^ In the present work, we have developed a series of novel terpolymer‐based AEIs by introducing bulky cyclohexyl groups, C6, into those partially fluorinated copolymers^[^ ^39^, ^40^ ^]^ expecting that the C6 groups should produce large free volume in the membranes to suppress excess swelling and exhibit high gas permeability.^[^ ^41^ ^]^ The low target IECs (≤2.2 mequiv g^−1^) were set to further enhance binding strength to the Ni_0.8_Co_0.2_O anode catalysts. The structure‐property relationship of the AEIs was thoroughly investigated to find out the optimum C6 composition. The optimized AEI was applied to AEMWEs as an anode binder to evaluate the performance and in situ durability.

## 2 Results and Discussion

### 2.1 Synthesis, Quaternization, and Characterization

**Scheme 1:**
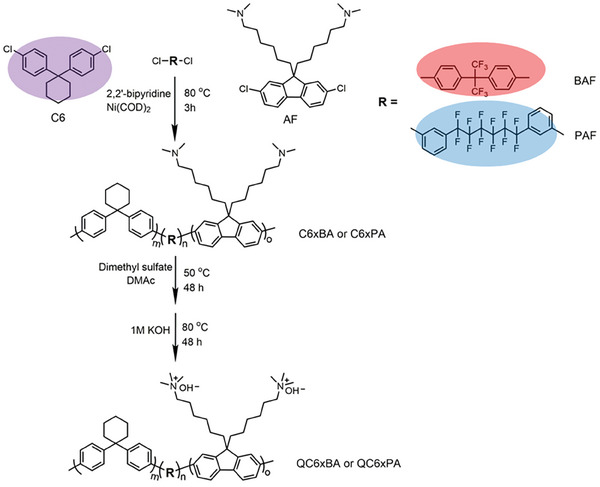
Synthesis of C6xBA and C6xPA and quaternized QC6xBA and QC6xPA.

The designed hydrophobic monomer with bulky cyclohexyl group, C6, was synthesized by acid‐catalyzed condensation and Sandmeyer reactions (Scheme S1, Figures S1 and S2, Supporting Information). A series of terpolymers containing two hydrophobic and one hydrophilic components were synthesized and quaternized as shown in **Scheme** **1**, where QC6xBA contained hexafluoroisopropylidende biphenylene (BAF) groups and QC6xPA contained perfluorohexylene biphenylene (PAF) groups. By Ni(0)‐mediated coupling reaction, terpolymers (C6xBA and C6xPA) were synthesized where x (= 0, 17, 33, 50) represents the molar composition of C6 in the hydrophobic components. The target IEC of the quaternized terpolymers was set at high (2.1 or 2.2 mequiv g^−1^) or low (1.6 mequiv g^−1^) value by adjusting the feed hydrophilic monomer composition (**Table** **1**). C6xBA and C6xPA terpolymers were obtained in high yields (>90%) with reasonably high molecular weight (*M*_n_ = 9.1−31.3 × 10^3^, *M*_w_ = 36.7−122.0 × 10^3^). Notably, the molecular weight of C6xPA decreased as decreasing the PAF composition or increasing the C6 composition, indicating that the reactivity of C6 was lower than that of PAF. The tendency was more pronounced for the lower IEC terpolymers because of the smaller content of the hydrophilic component. In the series of C6xBA terpolymers, the molecular weight was not related to the hydrophobic composition. The highest molecular weight was achieved with C6_33_BA‐1.6 (*M*_n_ = 31.3 × 10^3^, *M*_w_ = 117.0 × 10^3^) and C6_50_BA‐2.1 (*M*_n_ = 15.0 × 10^3^, *M*_w_ = 105.0 × 10^3^) among the C6xBA terpolymers with 1.6 and 2.1 mequiv g^−1^ of the target IECs, respectively. ^1^H and ^19^F NMR spectra (Figures S3 and S4, Supporting Information) confirmed the supposed chemical structures of C6xBA and C6xPA terpolymers, where the proton peaks were well‐assigned. In the ^1^H NMR spectra, the signal at 2.35 ppm was assigned to the methylene groups (**H_8_** in Figure S3 and **H_10_** in Figure S4, Supporting Information) in the cyclohexyl unit. The signal at 0.71 ppm was assignable to the methylene groups (**H_12_** in Figure S3 or **H_14_** in Figure S4, Supporting Information) in the alkyl chain. The calculated terpolymer compositions from the peak integrals were in good accordance with the target terpolymer compositions. In the ^19^F NMR spectra, the peak at −63.5 ppm (Figure S3, Supporting Information) was assigned to the CF_3_ groups in the BAF unit, and the peaks of the perfluorohexylene groups in the PAF unit appeared in the range from −110.4 to −121.1 ppm (Figure S4, Supporting Information).

**Table 1:** Composition, IEC, molecular weight, and membrane‐farming capability of the terpolymers.

Terpolymer	x^a)^	m: n: o^b)^	IEC [mequiv g^−1^]		*M*_n_ ^c)^ [kDa]	*M*_w_ ^d)^ [kDa]	Membrane‐forming capability
			target	titrated^c)^			
QC6_0_BA	0	0: 1: 0.39	1.6	1.50	11.5	66.9	○
QC6_17_BA	17	1: 5: 2.27		1.56	18.9	61.4	○
QC6_33_BA	33	1: 2: 1.09		1.53	31.3	117.0	○
QC6_50_BA	50	1: 1: 0.70		–	10.6	36.7	×
QC6_0_PA	0	0: 1: 0.59	1.6	1.57	15.8	122.0	○
QC6_17_PA	17	1: 5: 3.27		1.66	9.6	108.0	○
QC6_33_PA	33	1: 2: 1.49		1.64	9.1	70.4	○
QC6_50_PA	50	1: 1: 0.90		1.65	11.0	37.4	○
QC6_0_BA	0	0: 1: 0.65	2.1	1.95	10.2	82.6	○
QC6_17_BA	17	1: 5: 3.72		1.99	10.8	62.3	○
QC6_50_BA	50	1: 1: 1.14		1.88	15.0	105.0	○
QC6_0_PA	0	0: 1: 1.07	2.2	1.99	10.6	64.9	○
QC6_17_PA	17	1: 5: 5.90		2.12	13.6	64.0	○
QC6_50_PA	50	1: 1: 1.62		2.14	12.6	60.6	○

^a)^ the composition percentage of C6 in the hydrophobic segments, x = m × 100/(m + n);

^b)^ the feed ratio of C6, BAF or PAF, and AF;

^c)^ IEC was measured by titration;

^d)^ molecular weight of C6xBA and C6xPA; ○bendable membranes; × fragile membranes.

**Figure 1:**
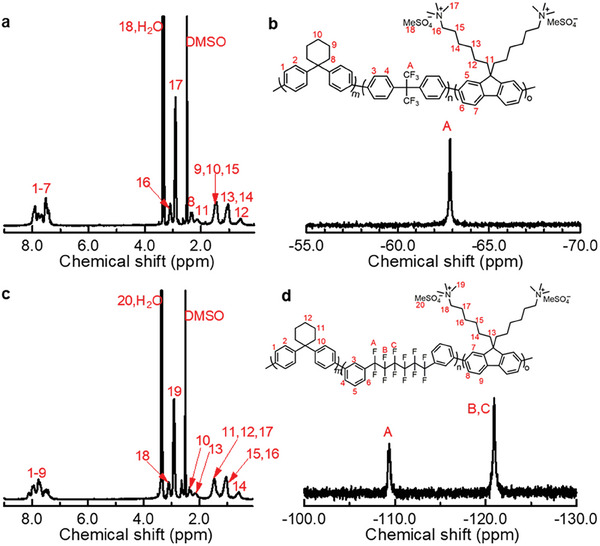
a) ^1^H NMR and b) ^19^F NMR spectra of QC6_50_BA‐2.1 terpolymer in DMSO‐*d_6_*. c) ^1^H NMR and b) ^19^F NMR spectra of QC6_50_PA‐2.1 terpolymer in DMSO‐*d_6_*.

The quaternization reaction was carried out using dimethyl sulfate (Me_2_SO_4_) in DMAc solution to obtain QC6xBA and QC6xPA in MeSO_4_^−^ form, where the color of the terpolymers changed from white to yellow. Compared with C6xBA and C6xPA, the quaternized QC6xBA and QC6xPA possessed better solubility not only in polar solvents such as DMAc but also in lower alcohols such as methanol. Good solubility in lower alcohols is highly preferable to prepare catalyst ink and enhance the ionomer‐catalyst adhesion for AEMWEs.^[^ ^42^ ^]^ In the ^1^H NMR spectra (**Figure** **1**), a new peak appeared at 3.37 ppm assignable to MeSO_4_^−^ groups as counter anion, and the peak of methyl groups attached to the nitrogen atoms shifted downfield from 2.11 to 2.90 ppm after the reaction, suggesting the successful quaternization of C6xBA and C6xPA terpolymers. By solution casting, QC6xBA and QC6xPA formed bendable and transparent membranes except for QC6_50_BA‐1.6 due to its lowest molecular weight. The titrated IECs were close to the target IECs, indicating a successful quaternization reaction.

### 2.2 Morphology

**Figure 2:**
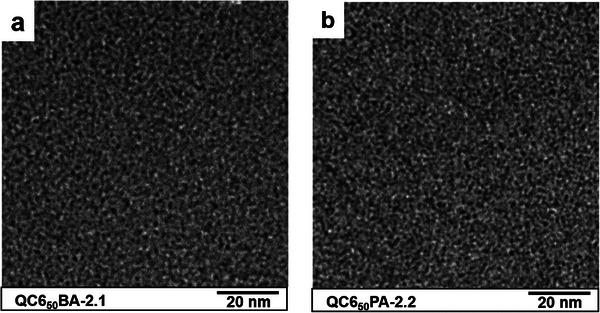
TEM images of membranes stained with PtCl_4_^2–^, a) for QC6_50_BA‐2.1 and b) for QC6_50_PA‐2.2.

Transmission electron microscopic (TEM) images were taken to investigate the phase‐separated morphology of the QC6xBA‐2.1 and QC6xPA‐2.2 membranes with different C6 composition (**Figure** **2** for x = 50 mol%, see Figure S5 for other compositions, Supporting Information). Bright regions were composed of the hydrophobic polymer backbone, while dark domains were composed of the hydrophilic ammonium groups and their aggregates. Due to the similar IEC values, the membranes showed similar morphology. For the quantitative discussion, domain sizes were averaged and plotted as a function of the C6 composition in Figure S6 (Supporting Information). In the series of QC6xBA‐2.1 membranes, the hydrophobic domains (1.42–1.66 nm) were slightly larger than the hydrophilic domain sizes (1.04–1.21 nm). Those domain sizes were unlikely to be dependent on the C6 composition. The hydrophobic domains (1.50–1.69 nm) were also slightly larger than the hydrophilic domain sizes (0.95–1.18 nm) in the series of QC6xPA‐2.2 membranes. It is noted that the hydrophilic domains became larger and the hydrophobic domains became smaller as increasing the C6 composition. For both series, the effect of the C6 composition on the membrane morphology was rather minor.

### 2.3 Water Uptake and OH^−^ Conductivity

**Figure 3:**
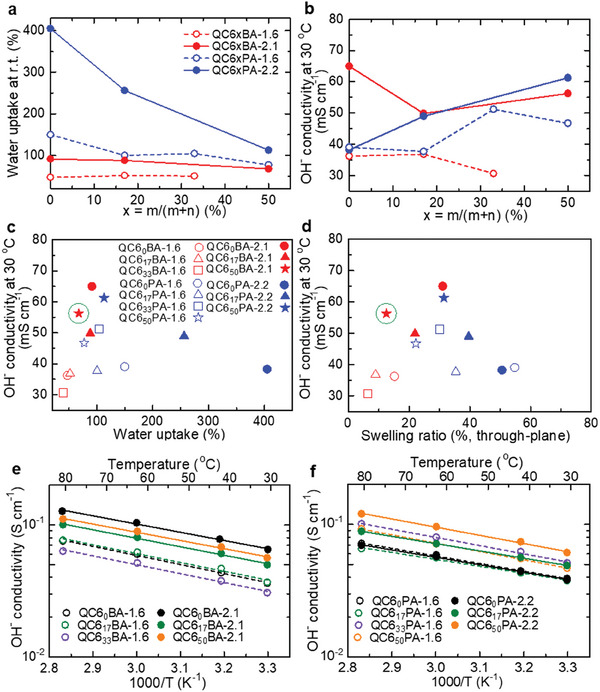
a) Water uptake at room temperature and b) OH^−^ conductivity at 30 °C in water as a function of C6 composition in the hydrophobic components. Correlation of c) water uptake, d) through‐plane swelling, and OH^−^ conductivity at 30 °C. Temperature dependence of OH^−^ conductivity of e) QC6xBA and f) QC6xPA in water from 30 to 80 °C.

Effects of C6 composition on the membrane properties were investigated. In **Figure** **3**a,b are plotted water uptake (at room temperature) and hydroxide ion conductivity (at 30 °C) as a function of the C6 composition. For both series of QC6xBA and QC6xPA membranes, the water uptake decreased as increasing the C6 composition. In particular, the water uptake of QC6xPA‐2.2 membranes significantly decreased from 405% (C6 = 0 mol%) to 113% (C6 = 50 mol%). The results imply that the bulky C6 groups increased hydrophobicity of the bulk membranes. Since the effect of C6 groups was so significant, other relevant parameters such as IEC (1.6 or 2.1 mequiv g^−1^) and the other hydrophobic components (PAF or BAF groups) impacted much less on the water uptake when C6 was 50 mol%. Similarly, the swelling of the QC6xBA and QC6xPA membranes became smaller as increasing the C6 composition, where swelling of QC6_50_BA‐2.1 decreased to 12.5% from 31.1% (through‐plane) and to 16.6% from 18.3% (in‐plane), and swelling of QC6_50_PA‐2.2 decreased to 31.5% from 50.6% (through‐plane) and to 24.0% from 71.7% (in‐plane) (Figure S7, Supporting Information). The results reveal that QC6_50_BA‐2.1 possessed the highest dimensional stability among the membranes with high IEC (2.1 or 2.2 mequiv g^−1^). QC6xPA‐2.2 and QC6xBA‐2.1 exhibited larger swelling than QC6xPA‐1.6 and QC6xBA‐1.6 due to higher IEC and water uptake.

As shown in Figure 3b, QC6xBA‐2.1 membranes showed higher hydroxide ion conductivity than that of QC6xBA‐1.6 membranes due to former's higher IEC value. In contrast, the conductivity of QC6xPA‐2.1 membranes was slightly higher than that of QC6xPA‐1.6 membranes. The smaller dependence of the conductivity of QC6xPA membranes on IEC value is because of the larger water absorbability, which caused the smaller apparent concentration of the ionic species (diluted with water molecules) in the bulk membranes. The effect of the C6 composition on the conductivity was well‐correlated with the water absorbability. The conductivity of QC6xPA membranes increased as increasing the C6 composition (or decreasing the water uptake), while that of QC6xBA membranes changed rather slightly with the C6 composition (64.9 and 56.2 mS cm^−1^ for QC6_0_BA‐2.1 and QC6_50_BA‐2.1, respectively, at 30 °C). The OH^−^ conductivity of QC6_50_BA‐2.1 was comparable with PBPA‐b‐BPP (0.15) (ca. 57 mS cm^−1^ at 30 °C), which was used as ionomer in PGM‐AEMWE using 1 m KOH aqueous solution at 80 °C.^[20^^]^ In addition, all membranes showed an increase in the OH^−^ conductivity with increasing temperature from 30 to 80 °C as shown in Figure S8 (Supporting Information), where the QC6xBA‐2.1 membrane displayed high OH^−^ conductivity over 100 mS cm^−1^ at 80 °C (127.1 and 110.5 mS cm^−1^ for QC6_0_BA‐2.1 and QC6_50_BA‐2.1, respectively). Figures 3c,d, and S9 (Supporting Information) compare the water uptake, through‐ and in‐plane swelling, and OH^−^ conductivity at 30 °C for all membranes investigated. Notably, the QC6_50_BA‐2.1 membrane having low water uptake and swelling ratio, and high OH^−^ conductivity seems to have overcome the trade‐off between the conductivity and dimensional stability, proving itself as a promising candidate for AEI.

Figure 3e,f shows the temperature dependence of the conductivity, where all QC6xBA and QC6xPA membranes showed Arrhenius‐type temperature dependence from 30 to 80 °C. The activation energies calculated from the slopes were ca. 12.8–13.2 kJ mol^−1^ for QC6xBA‐1.6, ca. 11.8–12.3 kJ mol^−1^ for QC6xBA‐2.1, ca. 10.1–12.0 kJ mol^−1^ for QC6xPA‐1.6 and ca. 10.5–11.8 kJ mol^−1^ for QC6xPA‐2.2 (Table S1, Supporting Information). The results suggest that the QC6xBA and QC6xPA shared a similar anion conduction mechanism, independent of the components and composition of the hydrophobic groups.

### 2.4 Mechanical Properties

Dynamic mechanical analysis (DMA) was applied to evaluate the mechanical stability of QC6xBA‐2.1 and QC6xPA‐2.2 membranes in Cl^−^ form under variable relative humidity (at 80 °C) as shown in Figure S10a–c (Supporting Information) and variable temperature (at 60% RH) in Figure S10d–f (Supporting Information). In humidity dependence, storage modulus (E') decreased as increasing the humidity for all membranes. In particular, QC6xPA‐2.2 membranes showed larger loss due to the higher water absorbability compared to the QC6xBA‐2.1 membranes. Since the effect of the C6 composition on water uptake was minor in QC6xBA‐2.1 membranes, QC6_0_BA‐2.1 and QC6_50_BA‐2.1 membranes were very similar in E' and its humidity dependence. However, due to the obvious decrease of water uptake in QC6xPA‐2.2 membranes after introducing C6 composition, the loss of E' and its humidity dependence in QC6_50_PA‐2.2 were significantly mitigated, compared with those in QC6_0_PA‐2.2. In temperature dependence, QC6_0_BA‐2.1 and QC6_50_BA‐2.1 both exhibited a slight change of E' and no transition in E″ curves, illustrating that they were not sensitive to temperature. In contrast, QC6_0_PA‐2.2 showed an obvious decrease in E', and an increase in E″ and tanδ from ca. 60 °C. The small variation of E', E″ and tanδ in QC6_50_PA‐2.2 indicated that introducing C6 groups was advantageous in bettering the thermal stability of the terpolymers.

### 2.5 Gas Permeability

**Figure 4:**
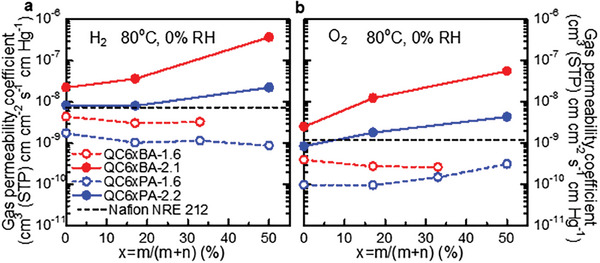
Gas permeability of QC6xBA and QC6xPA membranes as a function of C6 composition at 80 °C and 0% RH.

The gas permeability (H_2_ and O_2_) of QC6xBA and QC6xPA membranes was measured at 80 °C and dry conditions (0% RH) and plotted as a function of the C6 composition in **Figure** **4**. At any conditions investigated, QC6xBA membranes exhibited higher gas permeability than that of QC6xPA membranes, indicating that bulky BAF groups would effectively produce larger free volume in the membranes for gas permeation. For the membranes with lower IEC (1.6 mequiv g^−1^), the effect of the C6 composition was not significant and the gas permeability decreased slightly as increasing the C6 composition except for the O_2_ permeability of QC6xPA‐1.6; oxygen permeability increased by 3.2 times from C6 = 0 to C6 = 50 mol%. The higher IEC (2.1 or 2.2 mequiv g^−1^) membranes showed much higher gas permeability which was more dependent on the C6 composition. Similar to the BAF groups, C6 groups also contributed to the large free volume formation.^[^ ^41^, ^43^ ^]^ For QC6xPA‐2.2 membranes, H_2_ and O_2_ permeability increased by 2.7 and 5.2 times from C6 = 0 to C6 = 50 mol%. Effect of the C6 groups was more pronounced for QC6xBA‐2.1 membranes; H_2_ and O_2_ permeability increased by 16.6 times and 22.3 times, respectively, from C6 = 0 to C6 = 50 mol%. Overall, QC6_50_BA‐2.1 was the most gas permeable, with the permeability of 3.75 × 10^−7^ (H_2_) and 5.64 × 10^−8^ cm^3^ (STP) cm cm^−2^ s^−1^ cm Hg^−1^ (O_2_). For reference, the gas permeability of QC6_50_BA‐2.1 was 50.7 times and 46.6 times higher for H_2_ and O_2_ compared with that of Nafion NRE 212 membrane, which is a well‐known benchmark proton conductive ionomer used for PEMWEs and PEMFCs.

### 2.6 Alkaline Stability

**Figure 5:**
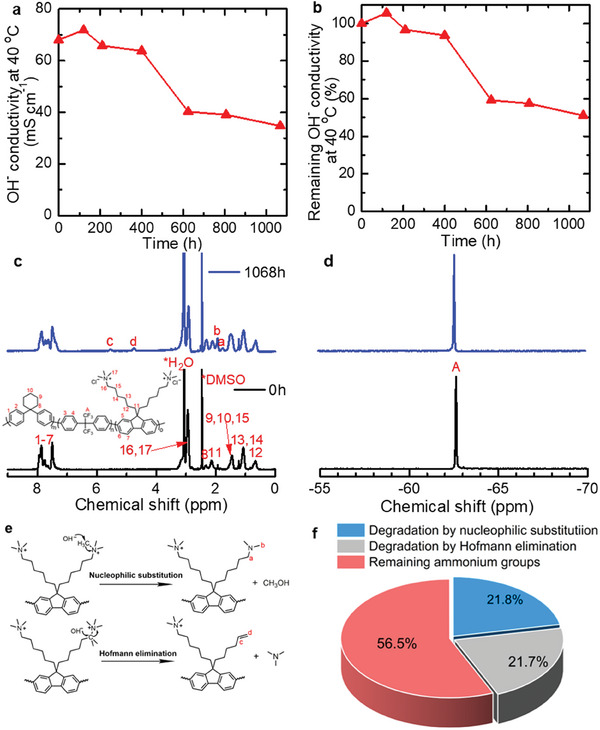
Alkaline stability of QC6_50_BA‐2.1 membrane in 8 m KOH at 80 °C. a) OH^−^ conductivity and b) remaining OH^−^ conductivity at 40 °C; c) ^1^H and d) ^19^F NMR spectra of QC6_50_BA‐2.1 before and after 1068 h alkaline stability test; e) possible degradation mechanisms of the ammonium groups in QC6_50_BA‐2.1 membrane; f) degree of alkaline decomposition in two pathways.

The alkaline stability of QC6_50_BA‐2.1 membrane was scrutinized by subjecting a sample to 8 m KOH aqueous solution at 80 °C where the sample was taken out at 100 or 200 h‐interval and soaked into degassed pure water to monitor the OH^−^ conductivity at 40 °C (**Figure** **5**a,b). After 1068 h, the QC6_50_BA‐2.1 membrane lost 49% of the OH^−^ conductivity from 68.0 to 34.8 mS cm^−1^. For comparison, the BAF‐QAF‐2.4 membrane without C6 groups lost 69% of the OH^−^ conductivity from 93.5 to 29.0 mS cm^−1^ under the same conditions.^[^ ^40^ ^]^ Somewhat better alkaline stability of QC6_50_BA‐2.1 would be due to its lower IEC, lower initial conductivity and the presence of C6 groups. Post‐test NMR analyses (Figure 5c,d) revealed that the integral ratio of the methylene protons in C6 (**H8**) to the aromatic protons (**H1**‐**H7**) in the ^1^H NMR spectra did not change as well as no changes in the ^19^F NMR spectra, suggesting excellent alkaline stability of the polyphenylene‐based main chain. However, four new peaks appeared around 1.75, 1.94, 4.73, and 5.56 ppm indicating the decomposition of the ammonium groups. The probable degradation pathways are described in Figure 5e. The peaks at 1.75 (**Ha**) and 1.94 ppm (**Hb**) were ascribed to the methylene and methyl groups in the pendent alkyl chain after undergoing nucleophilic substitution. The peaks at 4.73 and 5.56 ppm were assignable to the olefin groups (─CH═CH_2_) generated from Hofmann elimination. The integrals in the ^1^H NMR spectra enabled us to calculate the degree of degradation in two pathways, 21.8% nucleophilic substitution and 21.7% Hofmann elimination (Figure 5f). The remaining ammonium groups were estimated to be 56.5%, which was in good agreement with 51.2% remaining OH^−^ conductivity.

The post‐test QC6_50_BA‐2.1 membrane was subjected to the tensile properties test and compared with the pristine one as shown in Figure S11 and Table S2 (Supporting Information). The post‐test QC6_50_BA‐2.1 membrane exhibited comparable maximum stress (33.8, 32.7 MPa for the pristine sample) and lower maximum strain (49.7%, 95.1% for the pristine sample), presumably because the decomposition of the ammonium groups reduced the hydrophilicity of the post‐test membrane, resulting in smaller absorption of water to act as a plasticizer.

### 2.7 Water Electrolysis Performance

**Figure 6:**
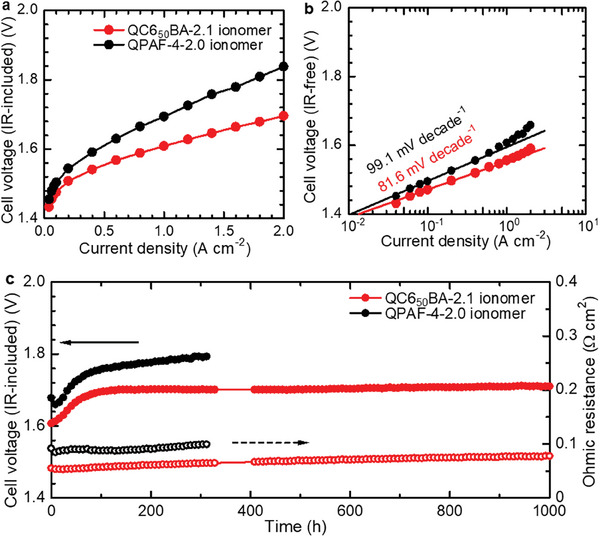
AEMWE performance of QC6_50_BA‐2.1 cell using QC6_50_BA‐2.1 as anode ionomer and QPAF‐4‐2.0 cell using QPAF‐4‐2.0 as anode ionomer at 80 °C with 1 m KOH aqueous solution. a) IR‐included IV curves, b) IR‐free Tafel plots, and c) in situ durability at a constant current density of 1.0 A cm^−2^. Test conditions: QPAF‐4‐2.0 as cathode ionomer, QPAF‐4‐1.5 (50 µm) as AEM, Ni_0.8_Co_0.2_O (2.0 mg cm^−2^) and Pt/C (1.0 mg cm^−1^).

Due to the excellent OH^−^ conductivity, dimensional stability, mechanical properties, alkaline stability, and high gas permeability, QC6_50_BA‐2.1 ionomer was selected as a binder in the anode catalyst layer. For comparison, another water electrolysis cell using QPAF‐4‐2.0 (same structure with QC6_0_PA) ionomer with similar IEC was also evaluated, where its structure as shown in Figure S12 (Supporting Information). Note that the same QPAF‐4‐2.0 ionomer was used in the cathode catalyst layer and the same QPAF‐4‐1.5 membrane (50 µm thick) was used for both cells. In **Figure** **6**a, the onset voltage was 1.43 and 1.45 V for QC6_50_BA‐2.1 and QPAF‐4‐2.0 cells, respectively, which was comparable to that (ca. 1.45 V) obtained for Ni_0.8_Co_0.2_O catalyst via rotating disk electrode in 1 m KOH aqueous solution.^[^ ^44^ ^]^ Obviously, the QC6_50_BA‐2.1 cell exhibited much better electrolysis performance than the QPAF‐4‐2.0 cell. The voltage efficiency was 76.4% and 73.2% at 1.0 A cm^−2^ for QC6_50_BA‐2.1 and QPAF‐4‐2.0 cells, respectively. At the same current density, the ohmic resistance of QC6_50_BA‐2.1 cell (0.055 Ω cm^2^) was 37% lower than that of QPAF‐4‐2.0 cell (0.087 Ω cm^2^), indicating better compatibility with the membrane presumably because of better dimensional stability and high conductivity. As increasing the current density from 1.0 to 2.0 A cm^−2^, the cell voltage increased from 1.61 to 1.69 V for the QC6_50_BA‐2.1 cell and from 1.69 to 1.84 V for the QPAF‐4‐2.0 cell. It is noted that at the same cell voltage of 1.69 V, the QC6_50_BA‐2.1 cell exhibited two times higher current density (2.0 A cm^−2^) than that of QPAF‐4‐2.0 (1.0 A cm^−2^), probably because highly gas permeable QC6_50_BA‐2.1 ionomer mitigated gas transport overpotential in the anode catalyst layer. For comparison, QC6_50_BA‐2.1 possessed 66.4 times and 572.6 times higher O_2_ permeability than that of QC6_0_PA‐2.2 and QC6_0_PA‐1.6, which had the same chemical structure with QPAF‐4‐2.0. Furthermore, the Tafel slope of QC6_50_BA‐2.1 cell in the IR‐free cell voltage (Figure S13, Supporting Information) was 81.6 mV decade^−1^ and the voltage deviated only slightly from the Tafel line even at 2.0 A cm^−2^ (Figure 6b). For the QPAF‐4‐2.0 cell, Tafel slope was larger (99.1 mV decade^−1^) and the cell voltage deviated more as increasing the current density. The results also suggest that QC6_50_BA‐2.1 functioned better as an AEI in the anode catalyst layer showing better gas transport capability at high current density.

The in situ durability of the cells was tested at a constant current density of 1.0 A cm^−2^ at 80 °C for 1000 h (Figure 6c). Both cells showed an increase in the cell voltage within the initial 100 h probably because of the interfacial structural changes of the ionomers in the chronoamperometric conditions. For QPAF‐4‐2.0 cell, the measurement was terminated at 311 h, where the cell voltage reached at 1.80 V at an increased rate of 19.0 µV h^−1^ (from 100 to 311 h). In contrast, the cell voltage was nearly constant for QC6_50_BA‐2.1 cell after the initial increase and reached 1.71 V after 1000 h at a much smaller increase rate of 1.1 µV h^−1^ (from 100 to 1000 h), indicating high in situ durability of the anode binder. In most previous works, the cell voltage increase rate was higher than 100 µV h^−1^ even at lower current density (≤0.5 A cm^−2^) in the long‐term in situ durability test.^[^ ^31^, ^38^, ^45^, ^46^, ^47^, ^48^, ^49^, ^50^, ^51^ ^]^ QC6_50_BA‐2.1 cell retained lower ohmic resistance (at an increased rate of 22.0 µΩ cm^2^ h^−1^) compared to that of the QPAF‐4‐2.0 cell (28.9 µΩ cm^2^ h^−1^), suggesting that the membrane and the interface of the catalyst layer with the membrane were also durable. After 1000 h in situ durability test, IV performance was somewhat deteriorated. As shown in Figure S14 (Supporting Information), the cell voltage (1.85 V at 2.0 A cm^−2^) was higher than that of the pristine QC6_50_BA‐2.1 cell but still comparable to that of the pristine QPAF‐4‐2.0 cell. Overall, it turned out that the design of AEI with high gas permeability and dimensional stability improved significantly the performance and in situ durability of AEMWE, promising for practical commercialization.

## 3 Conclusion

Two series of quaternized terpolymers (QC6xBA and QC6xPA) containing different compositions of cyclohexyl (C6) groups and similar IECs were designed and successfully synthesized as electrode binders for AEMWEs. The terpolymers, having either hexafluorobiphenylene (BAF) or perfluorohexylene (PAF) groups, exhibited good solubility in high boiling solvents and lower alcohols, as well as good membrane‐forming capability except for QC6_50_BA‐1.6. The introduction of the bulky C6 groups was favored to form large free volumes in the resulting membranes, causing significantly high gas permeability. The effect was more pronounced as increasing the C6 composition for higher IEC (2.1 or 2.2 mequiv g^−1^) membranes; as increasing the C6 composition from 0 to 50 mol% for QC6xPA‐2.2, the H_2_ and O_2_ permeability at 80 °C increased by 2.7 and 5.2 times, respectively. Similarly, H_2_ and O_2_ permeability increased by 16.6 and 22.3 times, respectively, for QC6xBA‐2.1. In fact, QC6_50_BA‐2.1 exhibited 51 times (H_2_) and 47 times (O_2_) higher permeability than that of Nafion NRE 212 (commercial benchmark proton exchange membrane and ionomer). Introducing C6 groups was also effective in suppressing the dimensional changes in water and improving alkaline stability with little expense of OH^−^ conductivity; high hydroxide ion conductivity (34.8 mS cm^−1^) was obtained with QC6_50_BA‐2.1 membrane after the accelerated alkaline stability test. The water electrolysis cell using the highly gas‐permeable QC6_50_BA‐2.1 ionomer in the anode catalyst layer achieved high performance (1.61 V) and voltage efficiency (76.4%) at the current density of 1.0 A cm^−2^ at 80 °C. The lower Tofel slope of QC6_50_BA‐2.1 cell (81.6 V decade^−1^) than that of QPAF‐4‐2.0 cell (99.1 mV decade^−1^) suggested the former's higher gas transport capability, especially at a high current density. Accordingly, the QC6_50_BA‐2.1 cell (1.69 V) outperformed QPAF‐4‐2.0 cell (1.84 V) at high current of 2.0 A cm^−2^. The electrolysis cell was further investigated in the long‐term operation to prove 1000 in situ durability at 1.0 A cm^−2^ and 80 °C.

## 4 Experimental Section

### Materials

2,2‐bis(4‐chlorophenyl)hexafluoropropane (BAF), bis(3‐chlorophenyl)perfluorohexane (PAF), and (3,3′‐(2,7‐dichloro‐9H‐fluorene‐9,9‐diyl)bis(N,N‐dimethylpropan‐1‐amine) (AF) monomers were synthesized according to the previous reports.^[^ ^39^, ^40^, ^52^ ^]^ Aniline (TCI), cyclohexanone (TCI), sodium nitrite (Kanto Chemical), copper (I) chloride (>99%, Kanto Chemical), bis(1,5‐cyclooctadiene)nickel(0) (Ni(COD)_2_) (> 95%, Kanto Chemical), 2,2′‐bipyridine (>99%, Kanto Chemical), dimethyl sulfate (>99%, Kanto Chemical), potassium hydroxide (> 86.0%, Kanto Chemical), and other chemicals and solvents were used as received.

### Synthesis of 4,4′‐(cyclohexane‐1,1‐diyl)dianiline (monomer 1)

Excess aniline (330.0 mmol, 30 mL) was added into a solution of cyclohexanone (91.8 mmol, 9.00 g) in 33 mL of 35% hydrochloric acid in a 100 mL flask. After stirring for 24 h at 100 °C, the mixture was cooled down and then, its pH value was adjusted to be ca. 13 by adding NaOH. The obtained oily layer was separated, distilled to remove the unreacted aniline, and then further purified by silica gel column chromatography (hexane: ethyl acetate = 10: 1 and then 3: 1). Monomer 1 was obtained as pale yellow solid (4.7 g, 19% yield).

### Synthesis of 4,4′‐(cyclohexane‐1,1‐diyl)bis(chlorobenzene) (C6)

C6 monomer was synthesized by Sandmeyer reaction as follows. A solution of NaNO_2_ (9.0 mmol, 0.62 g) in 5 mL water was added dropwise to a 200 mL flask charged with monomer 1 (3.8 mmol, 1.01 g) and 6 m hydrochloric acid (10 mL) at 0 °C. After 30 min, a solution of CuCl (11.1 mmol, 1.12 g) in 12 m hydrochloric acid (7 mL) was added slowly at room temperature. After 24 h reaction, the mixture was extracted with chloroform and washed with brine several times. Then, the organic layer was evaporated to remove the solvent and the resulting crude product was purified by column chromatography (eluent: hexane). C6 monomer was obtained as a colorless solid (0.79 g, 68% yield).

Polymerization and quaternization reaction (C6xBA and C6xPA, where x denotes the composition percentage of C6 in the hydrophobic segments): A typical polymerization procedure for C6_50_BA with target IEC 2.1 mequiv g^−1^ is as follows. A 100 mL flask filled with C6 (1.3 mmol, 0.40 g), BAF (1.3 mmol, 0.49 g), AF (1.5 mmol, 0.73 g), 2,2′‐bipyridine (24.6 mmol, 3.86 g), and DMAc (13 mL) was stirred and heated to 80 °C under nitrogen atmosphere. Ni(COD)_2_ (12.3 mmol, 3.38 g) was added to the homogeneous mixture. After a 3 h reaction, the mixture was cooled down and poured into a 500 mL beaker charged with methanol (150 mL) and concentrated hydrochloric acid (150 mL) to precipitate the product. The crude product was collected by filtration, washed with concentrated hydrochloric acid, 0.2 m K_2_CO_3_ aq, and water, subsequently. After drying at 60 °C in a vacuum oven overnight, 1.28 g of C6_50_BA was obtained in 97.0% yield.

For the quaternization reaction, 1.1 g of C6_50_BA polymer was dissolved in 10 mL of DMAc to form a homogenous solution. Dimethyl sulfate (Me_2_SO_4_, 1.2 mL) was added dropwise to the solution. The quaternization reaction was conducted at 50 °C for 48 h. The mixture was poured into deionized water. The crude product as a precipitate was collected by filtration, washed with water twice and dried in a vacuum oven at 60 °C overnight. 1.36 g of QC6_50_BA polymer (MeSO_4_^–^ form) was obtained in 95.7% yield.

The polymerization of C6xPA and quaternization of QC6xPA were similar, where PAF replaced BAF as another hydrophobic segment.

### Membrane Preparation and Ion Exchange Reaction

A solution of 0.5 g of quaternized polymer in 10 mL DMAc was filtered and poured onto a 10 × 10 cm glass plate to evaporate solvent at 50 °C overnight. A transparent membrane (in MeSO_4_^–^ form) was peeled off from the glass plate.

The membranes (in MeSO_4_^–^ form) were immersed in 1 M KOH at 80 °C for 48 h or 3 M NaCl at r.t. for 48 h to obtain membranes in OH^−^ form or Cl^–^ form, respectively.

### Measurements

^1^H and ^19^F NMR spectra were measured with a JEOL JNM‐ECA/ECX500 spectrometer, where the used solvents were DMSO‐*d*_6_ or CDCl_3_ containing internal reference (tetramethylsilane, TMS). The molecular weight of the polymers was measured with gel permeation chromatography (GPC) equipped with a Shodex K‐805 L column and a Jasco UV 2077 detector (270 nm). CHCl_3_ containing 0.02 M triethylamine was used as eluent and standard polystyrene was used for calibration.

### Ion Exchange Capacity, OH^−^ Conductivity, Water Uptake

The ion exchange capacity (IEC) of the membranes was measured by titration. Dry membrane in Cl^−^ form was soaked in 0.2 m NaNO_3_ aqueous solution for 24 h to release Cl^−^ ions from the membrane. The released Cl^−^ ions were titrated with 0.01 m AgNO_3_ aqueous solution, where the indicator and pH adjusters were K_2_CrO_4_ and NaHCO_3_, respectively.

A 4‐probe conductivity cell equipped with a Solartron 1255B AC impedance apparatus was applied to measure the hydroxide ion (OH^−^) conductivity of the membranes, where the membrane was placed into a 100 mL beaker containing degassed ultra‐pure water. During the measurement, nitrogen was bubbled to avoid contamination of carbon dioxide from the environment. The temperature was set at 30, 40, 60, and 80 °C. The OH^−^ conductivity (S cm^−1^) was calculated as follows: σ = l/(A × R), where R is the ion‐conducting resistance, A and l are the cross‐sectional area and the electrode distance, respectively. Water uptake of the membranes (in OH^−^ form) was measured at r.t. in water.

### Mechanical Properties

The dynamic mechanical analyzes (DMA) of the membrane (in Cl^−^ form) was measured with an ITK DVA‐225 dynamic viscoelastic analyzer. Storage modulus (E', Pa), loss modulus (E″, Pa), and tan δ ( = E″/E') were measured at the varying temperatures at 60% RH and varying humidity at 80 °C.

### Gas Permeability

A GTR‐Tech 20XFYC gas permeation measurement apparatus was used to measure the hydrogen and oxygen permeability of the membranes. The concentrations of the permeated gases were quantified by a Yanaco G2700T gas chromatograph with a Porapak‐Q column and a thermal conductivity detector. The carrier gases for hydrogen and oxygen were argon and helium, respectively. The membrane sample was placed in the measurement cells equipped with a gas inlet/outlet. The test gas was supplied onto one side of the membrane, and the carrier gas was supplied onto the other side of the membrane. The same humidity conditions were applied to both test and carrier gases to ensure homogeneous wetting of the membranes.

The membrane was equilibrated until stable permeation data were obtained. The flow gas was sampled and subjected to the gas chromatograph to quantify the test gas permeated through the membrane. The gas permeation coefficient, Q (cm^3^ (STD) cm cm^−2^ s^−1^ cmHg^−1^), was calculated by the following equation: Q = 273/T × 1/A × B × 1/t × l × 1/(76‐P_H2O_), where T (K) is the absolute temperature, A (cm^2^) is the permeation area, B (cm^3^) is the volume of the permeated test gas, t (s) is the sampling time, l (cm) is the thickness of the membrane, and P_H2O_ (cmHg) is the water vapor pressure.

### Alkaline Stability

The alkaline stability of the membranes was measured in 8 m KOH aqueous solution at 80 °C for 1068 h. The OH^−^ conductivity of the membrane sample was monitored at 40 °C in degassed ultra‐pure water. The post‐test membranes were analyzed by NMR spectra and tensile properties test.

### Membrane Electrode Assembly (MEA) Preparation

Home‐made catalyst Ni‐Co oxide (Ni_0.8_Co_0.2_O),^[^ ^44^ ^]^ 5 wt.% QC6_50_BA‐2.1 ionomer in methanol and ultra‐pure water were used to prepare anode catalyst ink, where the weight ratio of ionomer to catalyst was 0.15. The prepared anode catalyst ink was sprayed onto one side of a QPAF‐4‐1.5 membrane (IEC = 1.5 mequiv g^−1^, thickness = 50 µm) to form a catalyst‐coated membrane (CCM) using a Nordson pulse‐swirl‐spray apparatus (PSS machine). The catalyst loading amount was 2.0 mg_cat_ cm^−2^. The porous transport layer (PTL) for the anode side was nickel PTL (Bekaert Co., Ltd.). For cathode catalyst ink, Pt/C (TEC10E50E, Tanaka Kikinzoku Kogyo, K. K.) and the weight ratio of ionomer (QPAF‐4‐2.0 with 2.0 mequiv g^−1^ of IEC) to carbon was 0.6. Using the same PSS machine, the prepared cathode catalyst ink was sprayed onto a gas diffusion layer (GDL, TGP‐H‐120, Toray Co., Ltd.) to form a gas diffusion electrode (GDE), where the catalyst loading amount was 1.0 mg_Pt_ cm^−2^. The active geometric area of MEA was 1.0 cm^2^. MEA was fabricated from CCM, nickel PTL, and GDE. A gasket (EPDM, 300 µm of thickness) and a nickel separator with a straight flow path were placed on both sides of MEA. Gold‐plated copper was used as the current collector plate. The assembled electrolysis cell was sealed by applying an 8.5 kgf cm^−2^ pressure.

### Anion Exchange Membrane Water Electrolysis (AEMWE) Performance and In Situ Durability

The performance and in situ durability of AEMWEs were measured by the electrochemical station (Netsuden ind. Co., Ltd) combined with a regulated direct current power supply (PWR 401L, Kikusui Electronics Corp. current range from 0 to 40 A). 1 m KOH aqueous solution was fed to both sides of the cell at 10 mL min^−1^ at 80 °C. After the cell was stabilized at 80 °C for 2 h, the MEA was activated by sweeping the current density from 0 to 1.0 A cm^−2^ twice. Subsequently, the *I–V* was measured from 0 to 2.0 A cm^−2^ twice, followed by performing the in situ durability test at a constant current density (1.0 A cm^−2^) and 80 °C. Every 8 h, the *I–V* curves were measured until the total in situ durability time reached 1000 h.

## Conflict of Interest

The authors declare no conflict of interest.

## Supporting information

Supporting Information

## Data Availability

The data that support the findings of this study are available from the corresponding author upon reasonable request.
