# Supplementary material for: High‐Performance Anion Exchange Membrane Water Electrolyzers Enabled by Highly Gas Permeable and Dimensionally Stable Anion Exchange Ionomers
Source: Adv Sci (Weinh). 2024 Jun 3;11(29):2402969. doi: 10.1002/advs.202402969 (PMC11304252; doi:10.1002/advs.202402969)
Supplement: Supplementary file 1 — Supporting Information [file ADVS-11-2402969-s001.docx]

Supporting Information

**High-performance anion exchange membrane water electrolyzers enabled by highly gas permeable and dimensionally stable anion exchange ionomers**

*Fanghua Liu, Kenji Miyatake*, Masako Tanabe, Ahmed Mohamed Ahmed Mahmoud, Vikrant Yadav, Lin Guo, Chun Yik Wong, Fang Xian, Toshio Iwataki, Makoto Uchida, Katsuyoshi Kakinuma*

**

**

**Scheme S1**. Synthesis of monomer C6.

**
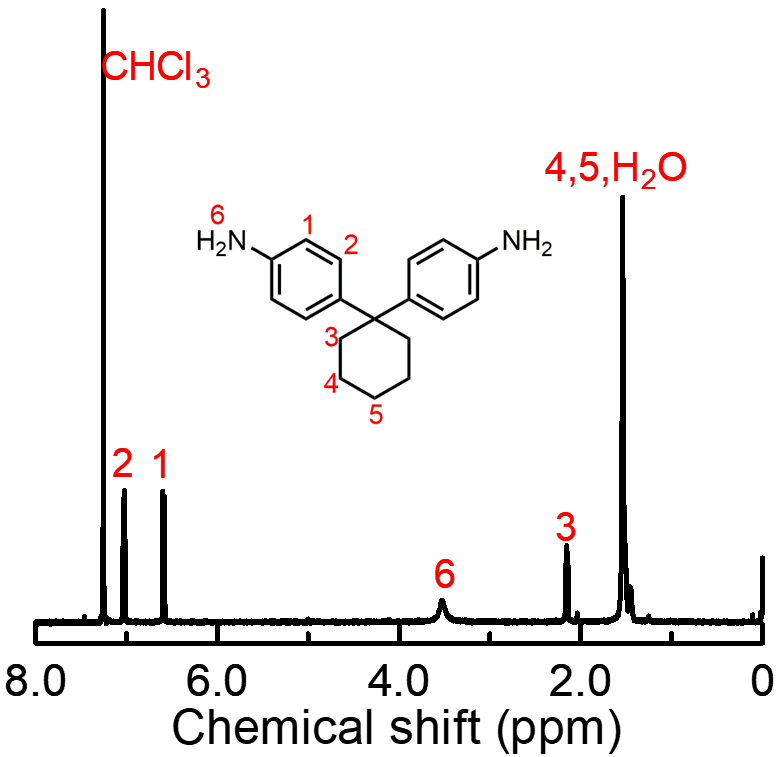
**

**Figure S1**. ^1^H NMR spectrum of monomer 1 in CDCl_3_.


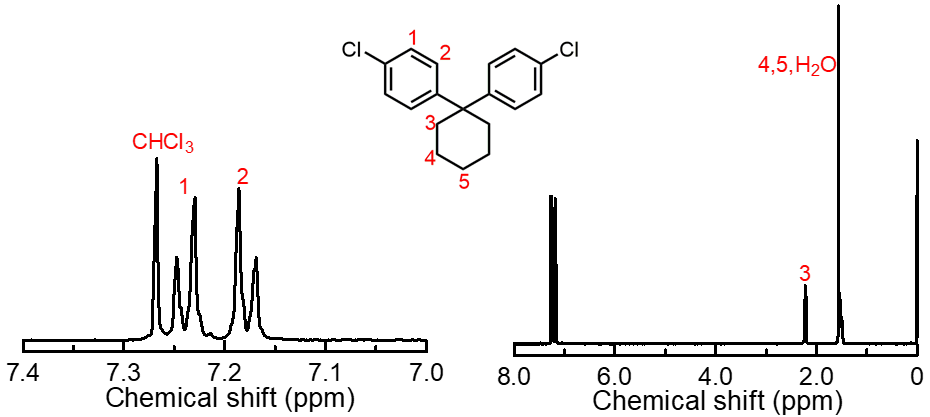


**Figure S2**. ^1^H NMR spectrum of C6 in CDCl_3_.


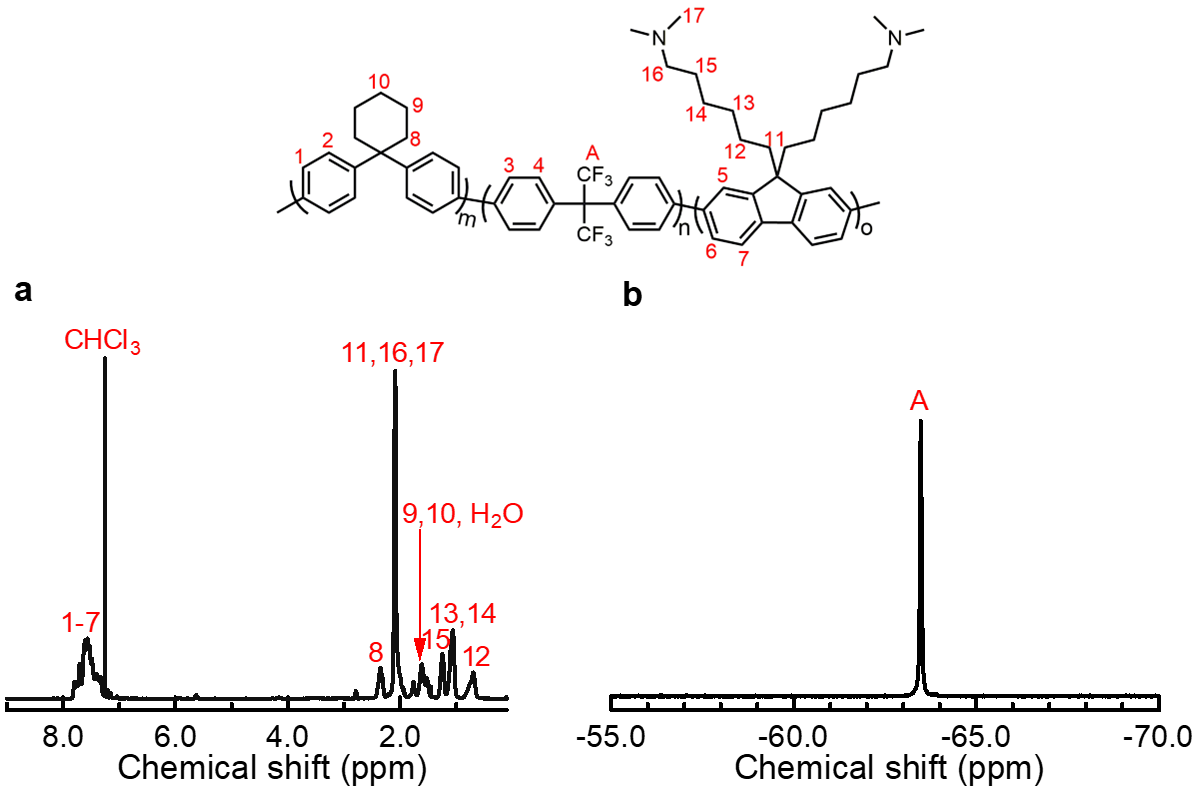


**Figure S3**. a) ^1^H NMR and b) ^19^F NMR spectra of C6_50_BA-2.1 terpolymer in CDCl_3_.


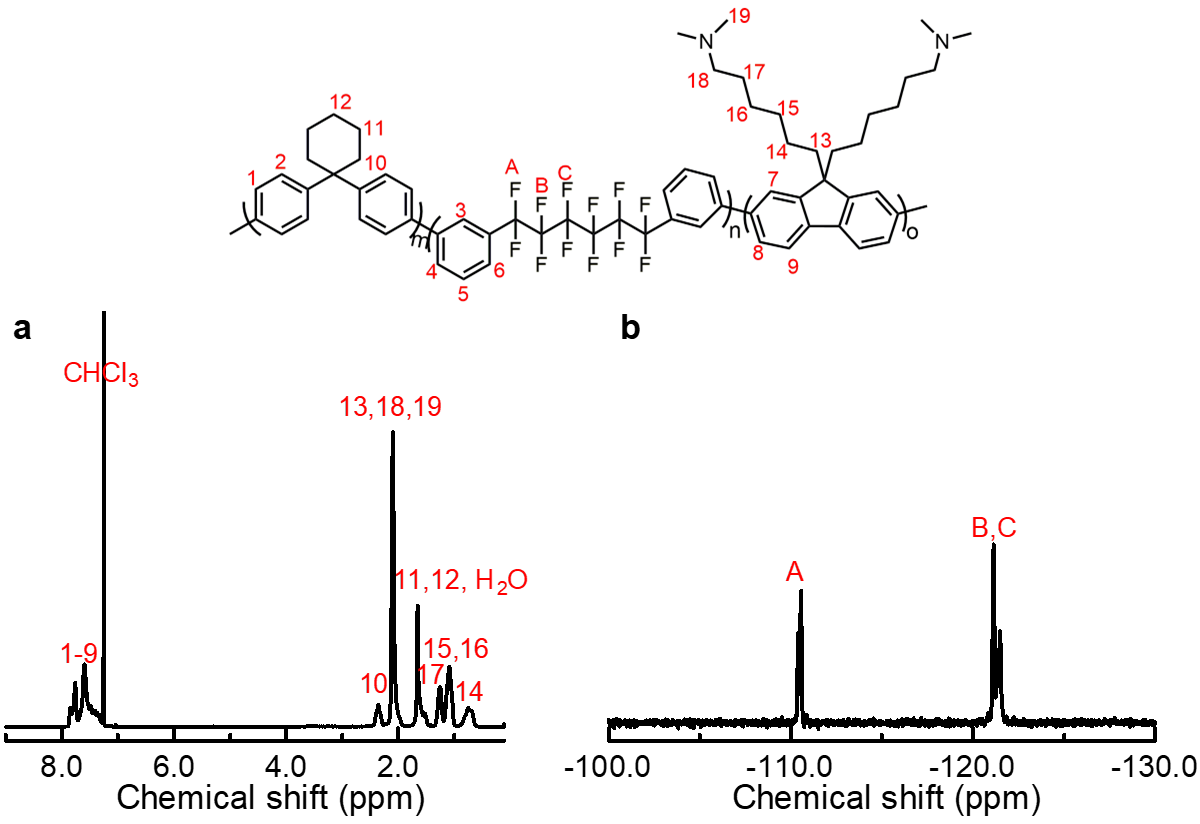


**Figure S4**. a) ^1^H NMR and b) ^19^F NMR spectra of C6_50_PA-2.1 terpolymer in CDCl_3_.


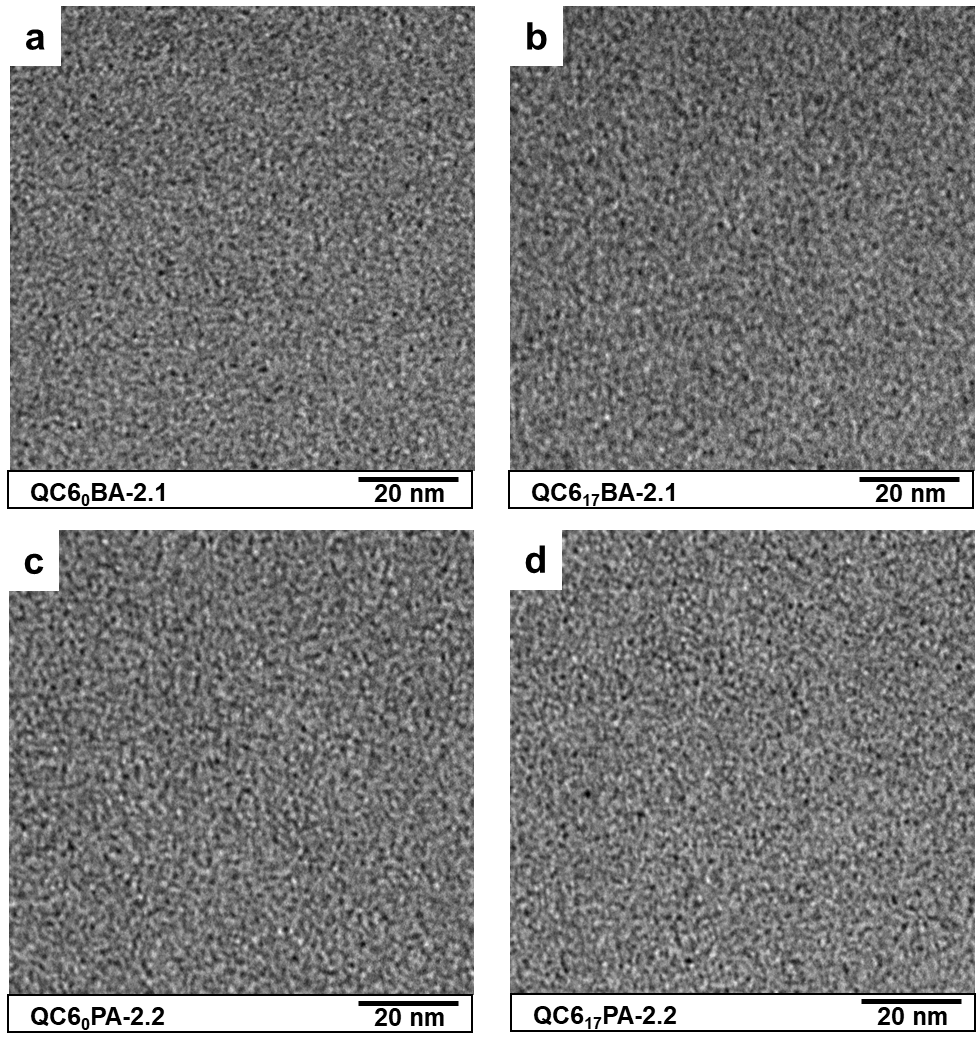


**Figure S5**. TEM images of the membranes stained with PtCl_4_^2–^, a-b) for QC6xBA-2.1 and c-d) for QC6xPA-2.2, where x= 0 and 17 mol%.

**Figure S6**. The dependence of hydrophobic and hydrophilic domain sizes on C6 composition.


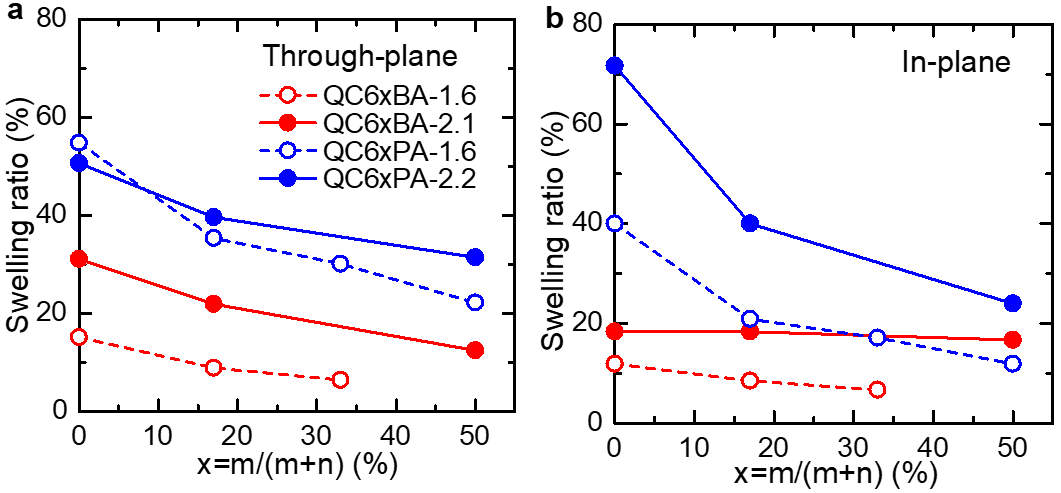


**Figure S7**. a) Through-plane and b) in-plane swelling of QC6xBA and QC6xPA at room temperature in water as a function of the C6 composition in the hydrophobic components.

**Figure S8**. OH^–^ conductivity of QC6xBA and QC6xPA membranes as a function of temperature.


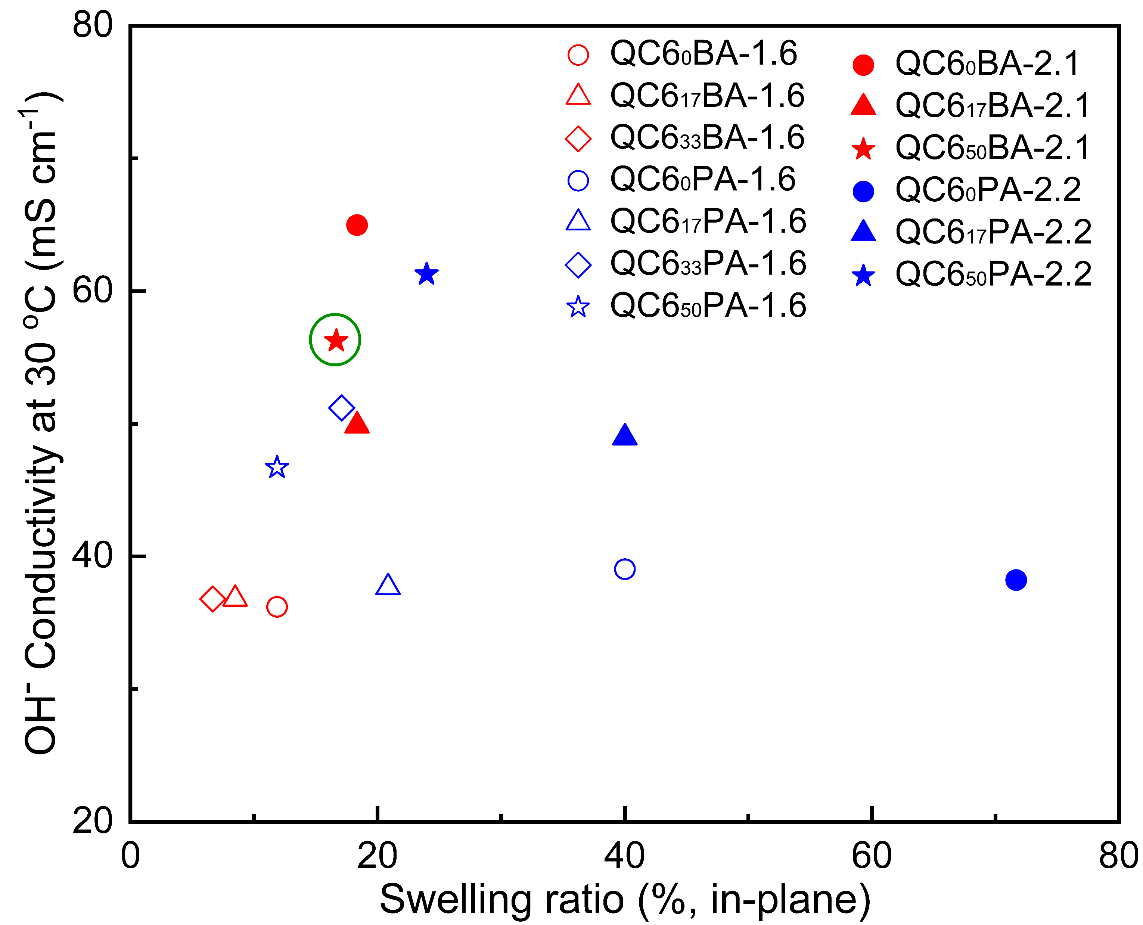


**Figure S9**. Correlation of in-plane swelling and OH^–^ conductivity at 30 ℃.


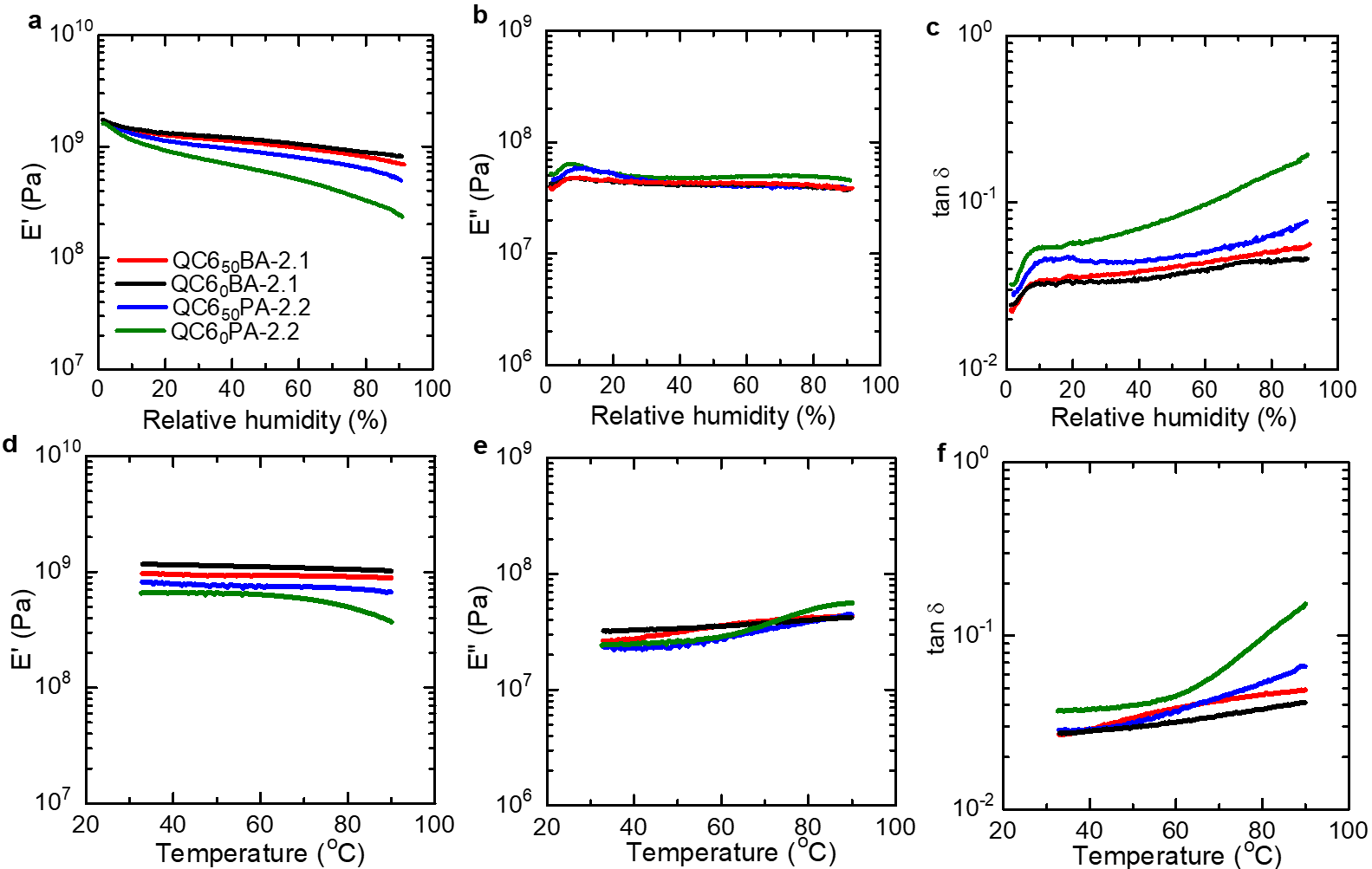


**Figure S10**. E’, E’’ and tan δ curves of QC6xBA-2.1 and QC6xPA-2.2 membranes as a function of relative humidity (a-c) at 80 °C and as a function of temperature (d-f) at 60% RH.

**Figure S11**. Stress versus strain curves of QC650BA-2.1 membrane before and after the alkaline stability test at 80 ℃ and 60% RH.





**Figure S12**. Structures of QC6_50_BA-2.1 and QPAF-4-2.0.

**Figure S13**. IR-included and IR-free I-V curves of QC6_50_BA-2.1 and QPAF-4-2.0 cell at 80 ℃ with 1 M KOH aqueous solution.

**Figure S14**. The AEMWE performance of QC6_50_BA-2.1 cell using QC6_50_BA-2.1 as an anode binder, before and after 1000 h *in-situ* durability.

**Table S1**. The activation energies of QC6xBA and QC6xPA.

| Membrane | Target IEC (mequiv g^-1^) | Activation energy (kJ mol^-1^) |
| --- | --- | --- |
| QC6_0_BA | 1.6 | 13.2 |
| QC6_17_BA |  | 12.8 |
| QC6_33_BA |  | 12.9 |
| QC6_0_BA | 2.1 | 11.9 |
| QC6_17_BA |  | 12.3 |
| QC6_50_BA |  | 11.8 |
| QC6_0_PA | 1.6 | 11.0 |
| QC6_17_PA |  | 10.1 |
| QC6_33_PA |  | 11.8 |
| QC6_50_PA |  | 12.0 |
| QC6_0_PA | 2.2 | 10.7 |
| QC6_17_PA |  | 10.5 |
| QC6_50_PA |  | 11.8 |

**Table S2**. The mechanical properties of QC6_50_BA-2.1 before and after the alkaline stability test at 80 ℃ and 60% RH.

| Membrane | Maximum stress (MPa) | Maximum strain (%) | Young’s modulus (GPa) |
| --- | --- | --- | --- |
| QC6_50_BA-2.1-0 h | 32.7 | 95.1 | 1.05 |
| QC6_50_BA-2.1-1068 h | 33.8 | 49.7 | 0.74 |
